# Variability in head computed tomography use for minor head injury after ground-level falls in the emergency department: A subanalysis of EPI-TC study

**DOI:** 10.1371/journal.pone.0334541

**Published:** 2026-01-02

**Authors:** Xavier Dubucs, Frederic Balen, Pierre-Hugues Cormicael, Axel Benhamed, Valérie Boucher, Éric Mercier, Sandrine Charpentier, Marcel Émond, IRU EPI-TC group

**Affiliations:** 1 Pôle médecine d’urgence, Centre Hospitalo-Universitaire Toulouse, Toulouse, France; 2 Centre d’Épidémiologie et de Recherche en santé des Populations, INSERM - UMR 1295, Université de Toulouse, Toulouse, France; 3 Centre de recherche du Centre Hospitalo-Universitaire de Québec-Université Laval, Québec City, Canada; 4 Centre d’excellence sur le vieillissement de Québec, Québec City, Canada; 5 VITAM – Centre de recherche en santé durable, Québec City, Canada; Centre Hospitalier de Troyes, FRANCE

## Abstract

**Objective:**

The aim of this study was to assess the variation in the use of head computed tomography (CT) scan in patients attending EDs with ground-level fall-related minor head injury. Secondary objectives were: i) to measure the prescription rate of appropriate head CT scan, ii) to identify patients’ and EDs characteristics associated with head CT scan prescription iii) to explore potential correlation between head CT scan use and traumatic intracranial hemorrhage (ICH) yield rate in this population.

**Materials and methods:**

This research was a planned sub-analysis of a cross-sectional prospective multicentric study performed in 63 EDs in France (EPI-TC study). Patients sustaining ground-level fall-related with minor head injury were included in this sub-analysis. The main outcome was head CT scan performed during ED stay. Variations in the use of head CT scan were described depending on each ED and French region. Multiple fixed effects mixed logistic regression model was performed to identify factors associated with head CT scan.

**Results:**

A total of 631 patients admitted for head injury after ground-level fall were included. Median age was 79 [63–88] years. A head CT scan was performed in 409 patients (64.8%, CI95% 61.0–68.5); 19.6% (CI95% 15.8–23.7) of them were appropriated; and 29 (7.1%, CI95% 4.8–10.0) showed a traumatic ICH. At regional level, head CT scan prescription rate ranged from 45.5% (CI95%: 24.4–67.8) to 84.6% (CI95% 54.5–98.1). Head CT scan use was not correlated with the yield rate of traumatic ICH. In multivariable analysis, preinjury antiplatelets (OR 29.2, CI95%: 12.2–69.9), anticoagulants (OR 69.9, CI95%: 20.0–243.9), syncope (OR 6.9, CI95%: 2.0–24.2), post-trauma amnesia (3.2, CI95%: 1.0–10.5) and post-trauma loss of consciousness (OR 5.6, CI95%: 2.0–15.9) were associated with head CT scan use.

**Conclusions:**

Head CT scan use in patient presenting to EDs with head injuries after ground-level falls is highly variable. High rate of head CT scan use is not correlated with high traumatic intracranial hemorrhage yield rate. The use of a clinical decision rule dedicated to this population would be suitable for harmonizing our practices.

## Introduction

Traumatic Brain Injury (TBI) is a common cause of admission to Emergency Departments (ED) and most of them are classified as mild TBI [1]. Non-contrast head computed tomography (CT) scan is the gold standard in TBI to detect traumatic Intracranial Hemorrhage (ICH). However, recent studies have shown an overuse of head CT scan up to 15%in case of mild TBI [2]. This is particularly significant given that repeated exposure to x-ray source radiation may be associated with an increased risk of cataracts and neoplasia [3,4]. It also represents a substantial financial burden for healthcare systems and may increase patients’ length of stay. A recent survey has reported a wide variation in the use of head CT scan in patients with mild TBI across many European countries [5]. Several environmental factors may be related to this variation such as the existence of national guidelines, their adherence as well as patients’ characteristics [5,6]. This is of the upmost importance to homogenize practices given that ground-level falls became the leading cause of head injury, especially in older patients [7]. Indeed, it has been shown that 40% of head CT scans performed in patients presenting minor head injury were related to ground-level fall [8]. Furthermore, recent literature has shown an increased number of older patients with TBI admitted to EDs. This trend is associated with a significant increase of CT scan in this population [9,10]. However, the assessment of recent clinical practices regarding head CT scan have only been evaluated using surveys and thus data from usual care setting are lacking [5,11]. In addition, the variation of head CT scan in patients with head injury only related to ground-level fall has not been carried out yet.

The aim of this study was to assess the variation in the use of head CT scan in patients attending EDs with ground-level fall-related minor head injury. Secondary objectives were: i) to measure the prescription rate of appropriate head CT scan, ii) to identify patients’ and EDs characteristics associated with head CT scan prescription iii) to explore potential correlation between head CT scan use and traumatic ICH yield rate in this population.

## Materials and methods

### Study design and settings

This was a planned sub-analysis of a prospective observational cohort study that collected patients’ data over a three-day period (from 06/03/2023 8:00 am to 09/03/2023 8:00 am, continuously H24) in March 2023 across 71 French EDs [12]. The study cohort comprised EDs across France that accepted an invitation from the *Initiative Recherche Urgence* (IRU; Emergency Research Initiative) network of the *Société Française de Médecine d’Urgence* (SFMU; French Society of Emergency Medicine) to participate in this study. The IRU is a research group that belongs to the French National Society of Emergency Medicine which includes more than 100 EDs in France. In this study, 12 of the 13 regions of Metropolitan France were represented (Auvergne-Rhône Alpes, Bourgogne-Franche-Comté, Bretagne, Centre – Val de Loire, Grand Est, Hauts-de-France, Normandie, Nouvelle Aquitaine, Occitanie, Pays de la Loire, Provence-Alpes-Côte-d’Azur, Île-de-France) and 3 of the 5 French departments overseas (La Réunion, Guadeloupe, Martinique).

### Participants

In this sub-analysis, only patients sustaining a ground-level fall-related with minor head injury (defined as Glasgow Coma Scale (GCS) score ≥13 upon arrival) were included. Patients with unknown head injury kinetic were excluded.

### Data collection and study variables

Upon admission to the ED, the physicians onsite gathered standardized data using the DoqBoard.com observational research platform. This included sociodemographic information, pre-injury use of antiplatelet and anticoagulant medications, fall precipitating factors, symptoms experienced after head injury, clinical examination at the ED and post-emergency outcome (discharge, hospital admission and in-hospital death). Urgent neurosurgical interventions occurring within 48 hours after admission were also reported.

### Outcome measures

The main outcome was non-contrast head CT scan performed during ED stay. The secondary outcome was appropriate head CT scan defined by the French National Guidelines published in 2022. Hence, head CT scan prescription was classified as appropriate if patients undergoing head CT scan showed intermediate or high risk of traumatic ICH [13]. Head CT scan prescription in mild TBI patients across French EDs is determined by the National Guidelines published in 2012 and updated in 2022 [13,14]. These guidelines recommend a head CT scan for mild TBI patients at intermediate risk (patients aged ≥65 years old with antiplatelet therapy, Glasgow Coma Scale (GCS) score <15 two hours after the trauma with a suspected intoxication, that sustained high-energy trauma, or with amnesia ≥30 minutes after the trauma) within 8 hours after being admitted to the ED. In patients presenting a high risk of traumatic ICH (hemostasis disorders, suspected basilar or cranial skull fracture, GCS < 15 two hours after the trauma without intoxication, > 1 vomiting episode, post-traumatic seizures, focal neurological deficit) a head CT scan is required within the first hour.

### Statistical analysis

Categorical variables were reported with their frequency, proportion (%) and 95% Confidence Interval (CI 95%), where appropriate. The description of quantitative variables was recorded through their median and interquartile ranges (IQ1–IQ3) and their mean and standard deviation (SD). Variations in the use of head CT head were described at the ED and at the regional level. In France, regional colleges of emergency physicians that offer annual training conferences and continuing education courses. These courses are not standardized at the national level. National guidelines must be followed by all emergency physicians, but there may be some variability in practices related to the regional training courses offered. La Réunion, Guadeloupe and Martinique were pooled as a unique overseas group of regions to reach a sufficient number of subjects. The prevalence of traumatic ICH was calculated among patients who underwent a head CT scan. The proportion of appropriate use of head CT scan was calculated based on the number of head CT scan prescriptions. The correlation between the prevalence of traumatic ICH yield and head CT scan prescription rates -both overall and appropriate use- was assessed using Spearman’s rank correlation coefficient. Bivariate analyses were performed to compare patients and ED characteristics to the use of head CT scan. These characteristics associated with the use of head CT scans with p-value < 0.2 were then included in a multiple fixed effects mixed logistic regression model. The dependent variable was the use of head CT scan at the patient’s level. Random intercepts were modeled per region and per ED sites within regions to allow varying baseline probability to use CT scans. In a first model building step, the association between the use of head CT scans and each patient-level and ED-level characteristics was modeled using mixed logistic regression with the mentioned random effects. A backward stepwise selection method was applied based on the area under the receiver operating characteristic curve (AUC). Missing data rates were reported for each variable and no data imputation was performed. All analyses were performed with Stata 17.0 (StataCorp, Texas, USA). Our results were reported using the STROBE statement.

### Ethics statement

According to French and European law on ethics, all included patients were informed that their anonymized data will be used for the study. The oral non-opposition of all patients was collected, and a written information notice was given to each included patient explaining the objectives of the study and the legislation explaining this research. This non-opposition was documented in the patient’s medical file. According to the French ethics and regulatory law (public health code) prospective studies based on the exploitation of usual care data should not be submit at an ethics committee (IRB), but they have to be declared or covered by reference methodology of the French National Commission for Informatics and Liberties (CNIL). Toulouse University Hospital signed a commitment of compliance to the reference methodology MR-004 of the French National Commission for Informatics and Liberties. After evaluation and validation by the data protection officer and according to the General Data Protection Regulation (Regulation EU 2016/679 of the European Parliament and of the Council of 27 April 2016), this study completing all the criteria, it is register in the register of data study of the Toulouse University Hospital (number’s register: RnIPH 2022–78) and cover by the MR-004 (CNIL number: 2206723 v 0). This study was approved by Toulouse University Hospital and confirms that ethical requirements were totally respected in the above report.

## Results

**Fig 1:**
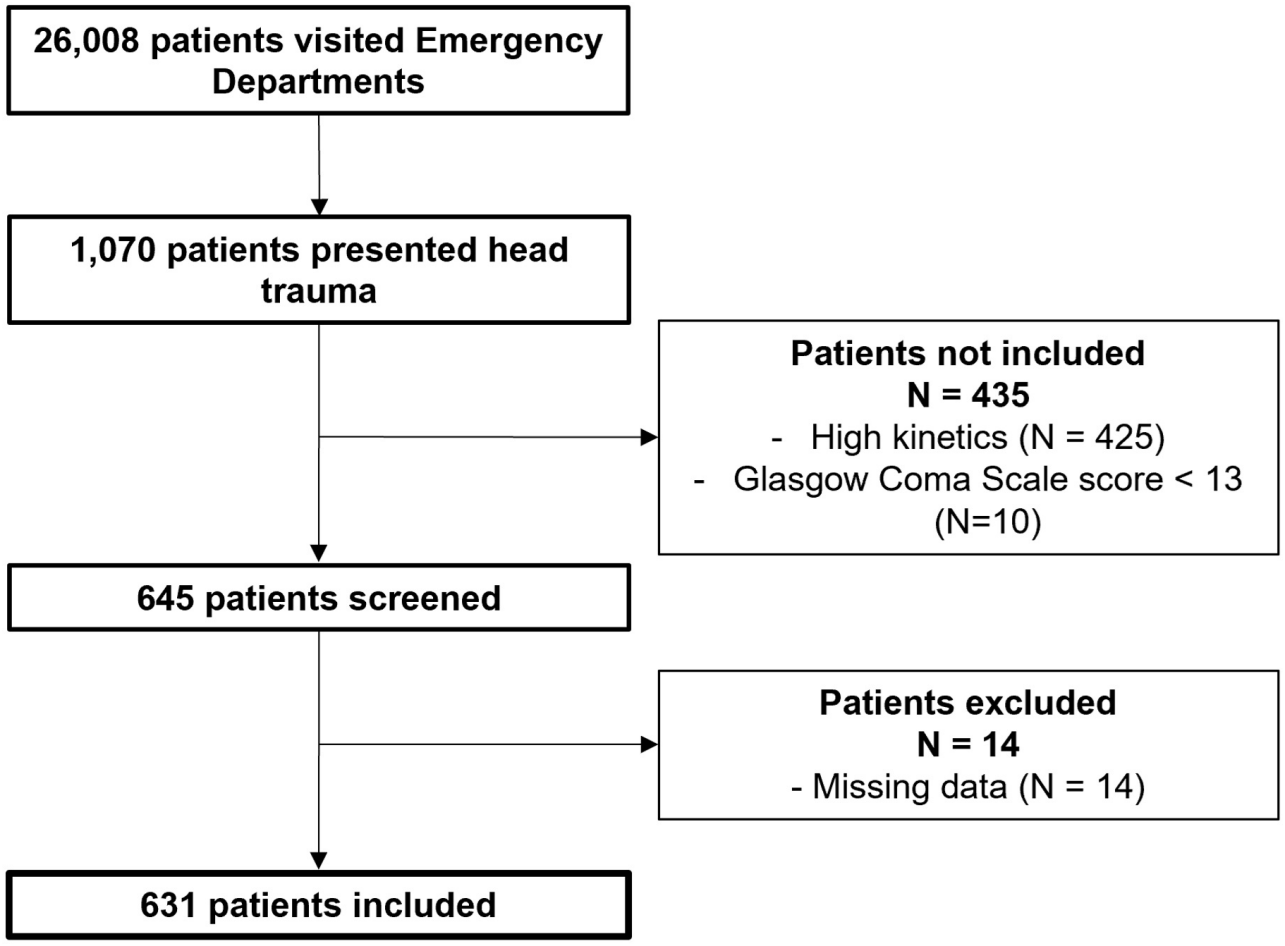
Flow chart.

A total of 631 patients admitted in one of the 63 participating EDs for head injury after ground-level fall were included (Fig 1). Median age was 79 years (63–88); environmental falls were the leading precipitating factor and more than half of patients (60.5%) had no post TBI symptoms. Baseline patients’ characteristics are displayed in Table 1. A head CT scan was performed in 409 patients (64.8%, CI95% 61.0–68.5) and a traumatic ICH was reported in 29 (7.1%, CI95% 4.8–10.0) of them. Among head CT scan prescriptions, 80 of them (19.6%, CI95% 15.8–23.7) were classified as appropriate. No significant correlation was found between overall head CT scan use (Spearman’s correlation coefficient: 0.22, p = 0.46) or appropriate head CT scan use (Spearman’s correlation coefficient: 0.06, p = 0.86) and traumatic ICH yield rate.

**Table 1:** Patients’ characteristics admitted to the Emergency Department with ground-level fall-related minor head injury.

	Overall N = 631	Missing value
**Age, median (IQ1-IQ3)**	79 (63-88)	
**Sex, female**	383 (60.7)	
**Place of residence**		5 (0.8)
** Community-dwelling**	483 (76.6)	
** Nursing Homes**	137 (21.7)	
** Homelessness**	6 (1.0)	
**Antiplatelets**	154 (24.4)	14 (2.2)
**Anticoagulants**	141(22.4)	15 (2.4)
**Post head injury symptoms**		54 (8.6)
** No symptom**	382 (60.5)	
** Amnesia**	39 (6.2)	
** Loss of consciousness**	69 (10.9)	
** Confusion**	72 (11.4)	
** Headache**	93 (14.7)	
** Seizure**	3 (0.5)	
** Vomiting**	37 (6.4)	
**Clinical findings at the ED**		
**Coma Glasgow Scale Score**		
** *15***	587 (93.0)	
** *14***	37 (5.9)	
** *13***	7 (1.1)	
**Visible head impact location**		6 (0.9)
** None**	155 (24.6)	
** Facial**	104 (16.5)	
** Frontal**	153 (24.3)	
** Temporal-parietal-occipital**	172 (27.3)	
** Multiple**	41 (6.5)	
**Focal neurological signs**	16 (2.5)	16 (2.5)
**Pupillary abnormalities**	6 (1.0)	24 (3.8)
**Basal skull fracture signs**	20 (3.2)	16 (2.5)
**Fall precipitating factor**		146 (23.1)
** Environmental**	323 (51.2)	
** Syncope**	45 (7.1)	
** Faintness or vertigo**	81 (12.8)	
** Alcohol intoxication**	29 (4.6)	
** Others**	7 (1.1)	

**Fig 2:**
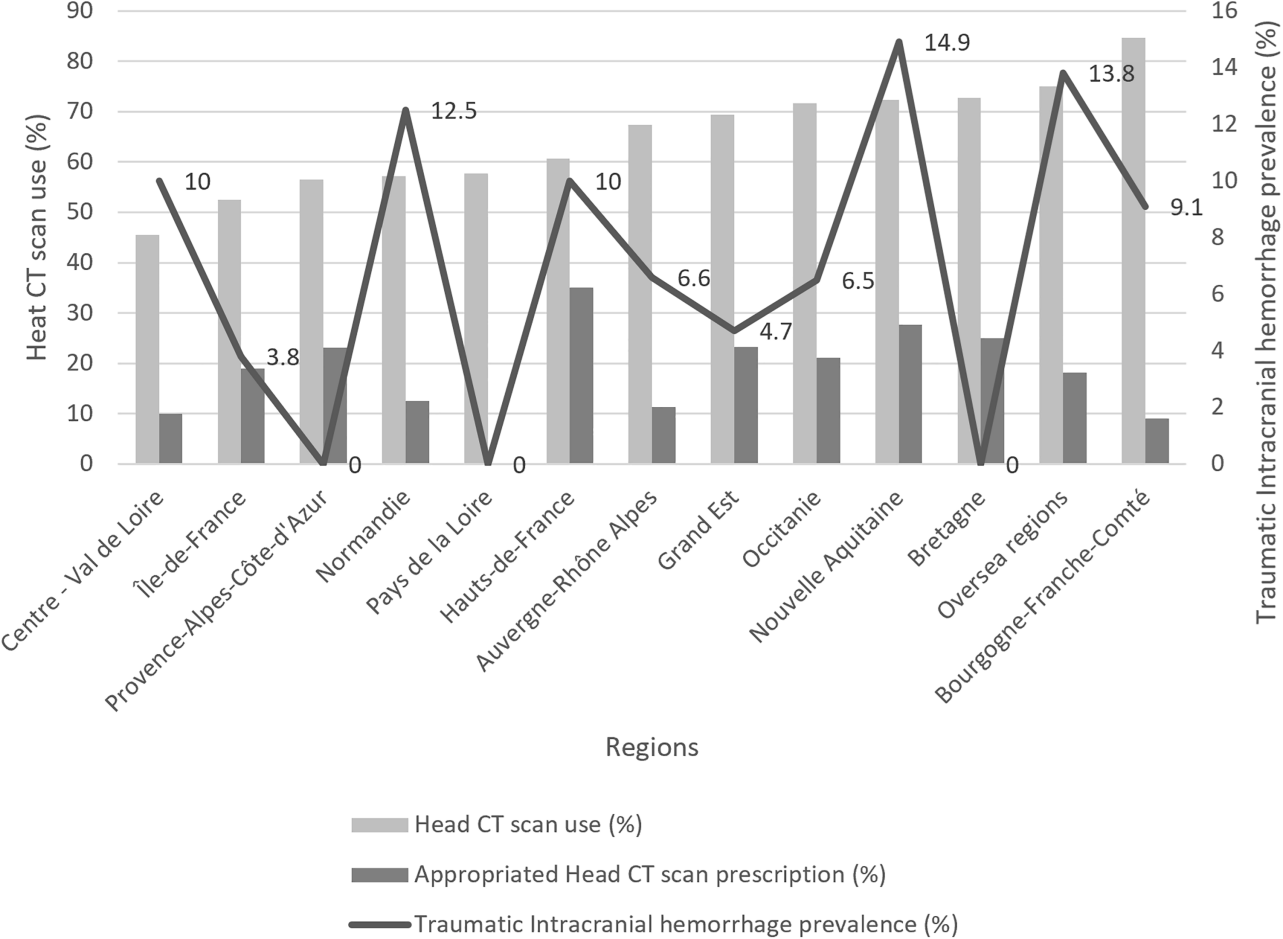
Traumatic intracranial hemorrhage yield rate according to head CT scan use at the regional level in patients presenting to Emergency Departments for head injury related to ground-level fall.

At the regional level, while the main patients’ characteristics were similar (Table 2), head CT scan prescription rates were statistically different (p = 0.02) ranged from 45.5% (CI95%: 24.4–67.8) to 84.6% (CI95% 54.5–98.1) (Fig 2). This variation in head CT scan prescription persisted in the subpopulation of patients with no neurological signs, those with no anticoagulants and those with no antiplatelets (Table 3). No statistically significant association was observed between the prevalence of traumatic ICH and regional distribution (p = 0.29) (Fig 2)

**Table 2:** Patients’ main characteristics according to French regions.

Provinces	Total	Type of institution, University Hospital (%)	Head CT scan use (%)	Age (IQ1-IQ3)	Sex, female (%)	Community-dwelling (%)	Antiplatelets (%)	Anticoagulants (%)	Coma Glasgow Scale score 15 (%)
Auvergne-Rhône Alpes	92	45 (48.9)	67.4 (CI95%: 56.8–76.8)	78 (63.5-87.5)	71.7	84.6	27.8	17.1	96.7
Bourgogne-Franche-Comté	13	6 (46.2)	84.6 (CI95%: 54.5–98.1)	87 (76-92)	53.9	69.3	23.1	30.8	100
Bretagne	22	21 (95.5)	72.7 (CI95%: 49.8–89.3)	83 (69-85)	68.2	81.8	28.6	33.3	95.4
Centre – Val de Loire	22	16 (72.7)	45.5 (CI95%: 24.4–67.8)	84.5 (64-91)	40.9	81.8	27.3	9.1	95.4
Grand Est	62	40 (64.5)	69.4 (CI95%: 58.7–66.7)	78 (69-87)	64.5	79	30.7	21.3	96.7
Hauts-de-France	33	0	60.6 (CI95%: 56.3–80.4)	86 (71-93)	75.8	69.7	17.8	27.6	96.9
Normandie	14	13 (92.9)	57.1 (CI95%: 28.9–82.3)	73 (66-86)	71.4	92.9	21.4	35.7	92.9
Nouvelle Aquitaine	65	27 (41.5)	72.3 (CI95%: 59.8–82.7)	82.5 (72-87)	49.2	79.7	24.6	30.8	86.2
Occitanie	106	59 (55.7)	71.7 (CI95%: 62.1–82.0)	77 (63-89)	57.5	72.4	24.8	31.4	89.6
Pays de la Loire	26	16 (61.6)	57.7 (CI95%: 36.9–76.6)	83 (59-88)	50	36	44	19.2	96.2
Provence-Alpes-Côte-d’Azur	46	10 (21.7)	56.5 (CI95%: 58.7–66.7)	88.5 (79-93)	60.9	71.7	21.7	23.9	95.7
Île-de-France	101	63 (62.4)	52.5 (CI95%: 42.3–62.5)	76 (61-88)	64.4	79	16	17.5	94.1
Oversea regions	29	29 (100)	75.0 (CI95%: 56.4–89.7)	68.4 (61-79)	41.3	96.5	17.9	3.4	79.3

**Table 3:** Head CT scan variation use in subpopulations at the regional level.

	Total	Head CT scan use prevalence	Region level Head CT scan use variation	ICH prevalence
**Minor head injury, GCS**^**a**^ **score 13–15**	631	64.8 (CI95%: 60.1–68.6)	45.5% (CI95%: 24.4–67.8) – 84.6% (CI95% 54.5–98.1)	7.10%
**Minor head injury, GCS 15, without focal neurological signs**	575	62.8 (CI95%: 58.7–66.7)	47.6% (CI95%: 25.7–70.2) – 84.6% (CI95% 54.5–98.1)	5%
**Minor head injury, GCS 15, neither focal neurological sign nor anticoagulants**	450	54.0 (CI95%: 49.3–58.7)	25.0% (CI95%: 3.2–65.1) – 77.8% (CI95% 40.0–97.2)	5.70%
**Minor head injury, GCS 15, neither focal neurological signs, anticoagulants nor antiplatelets**	323	40.6 (CI95%: 35.2–46.1)	11.1% (CI95%: 2.0–48.0) – 66.6% (CI95% 22.0–95.0)	4.60%

^**a**^GCS: Glasgow Coma Scale score; ICH: intracranial hemorrhage.

In bivariate analysis, ED’s characteristics were not associated with head CT scan use (S1 Table). S2 Table displayed bivariate analysis of patients’ characteristics regarding the use of head CT scan.

In multivariable analysis, preinjury antiplatelets (OR 29.2, CI95%: 12.2–69.9), anticoagulants (OR 69.9, CI95%: 20.0–243.9), syncope (OR 6.9, CI95%: 2.0–24.2), post-trauma amnesia (3.2, CI95%: 1.0–10.5) and post-trauma loss of consciousness (OR 5.6, CI95%: 2.0–15.9) were associated with the use of head CT scan (Table 4). Multivariable analysis of each subpopulation (patients with no neurological signs, those with no anticoagulants and those with no antiplatelets) are available in S3–S5 Tables (Subgroup multivariate analyses).

**Table 4:** Predictive factors associated with head CT scan use at the Emergency Department in patients with ground-level fall-related minor head injury.

	Odds ratio	CI 95%	p-value
Age, per year	1.0	0.9-1.1	0.26
Antiplatelets	29.2	12.2 - 69.9	<0.001
Anticoagulants	69.9	20.0 - 243.9	<0.001
Fall precipitating factor			
Syncope	6.9	2.0 - 24.2	<0.01
Faintness or vertigo	0.9	0.4 - 2.0	0.81
Alcohol intoxication	3.4	0.90 - 12.8	0.07
Others	0.8	0.1 - 8.8	0.84
Clinical findings at the ED			
Amnesia	3.2	1.0 - 10.5	0.05
Loss of consciousness	5.6	2.0 - 15.9	<0.01
Confusion	2.1	0.7 - 6.1	0.17

## Discussion

These results showed a wide variability in the use of CT scan among patients admitted to the ED after ground-level fall-related minor head injury. Our study suggests that this variation is not explained by patients’ characteristics. Several hypotheses may explain these findings. First of all, we assume that national guidelines on the use of head CT scan for patients admitted to ED with mild TBI are not always applied [13]. Assessing the implementation of clinical decision rules in clinical practice remains challenging [15]. For instance, in case of mild TBI, a recent study has shown that the implementation of the Canadian CT Head Rule was associated with a modest decrease in the use of head CT scan (absolute 5.3% reduction in the use of head CT scan) [16]. This decrease widely varied from one ED to another, ranging from +0.4% to −9%. While the performance of the Canadian CT Head Rule has been demonstrated, these results showed the difficulty of applying clinical decision rules to homogenize our clinical practices [16]. In addition, we can hypothesize that our study is premature in relation to the publication of these same guidelines in 2022. Not all emergency physicians have had time to get to know these new guidelines. Secondly, the median age of our cohort was 79 years (43–88). The risk stratification of traumatic intracranial hemorrhage is based on the collection of functional post-trauma signs (i.e.,: loss of consciousness, amnesia). However, nearly 30% of these older patients show cognitive impairment, making it difficult or impossible to collect these signs [17]. It is likely that physicians may indicate the realization of a head CT scan if there is any doubt. This may be at the source of a variation in practices, explaining the wide variability in head CT scan prescription. Moreover, in our study, ED characteristics (type of institution, number of admissions per day, onsite neurosurgery unit) were not associated with a variation in the number of prescriptions. The literature reports divergent results on this aspect. For instance, regarding the use of head CT scan among patients with suspected mild TBI, Ryu et al. showed that a head CT scan was more frequently prescribed in urban EDs than in rural ones [18].

Several patients’ characteristics were associated with head CT scan use. Antiplatelet and anticoagulant agents were the factors mostly associated with a head CT scan performance in our cohort. These results are probably explained by systematic indication, according to national guidelines, for head CT scan in anticoagulated patients [13]. In our cohort, almost half of the patients had preinjury antiplatelets or anticoagulants. These results are in line with those of the study performed by O’Brien et al. in which 70% of patients had preinjury antiplatelets or anticoagulants [19]. Recently, several studies have suggested that preinjury antiplatelets or anticoagulants medication may be not associated with traumatic ICH in patients after ground-level fall with head injury [20].

Furthermore, our study suggested that the rate of appropriate head CT scans in this population was low, and their use was not correlated with the traumatic ICH yield rate. These findings are in line with a study performed in 40 pediatric EDs in the US. Despite showing a large variability of head CT scan in minor head injured patients across pediatric EDs (ranged 19–58%), high rate of prescriptions was not associated with high rate of traumatic ICH [21].

Taken together, these results should incite us to use a clinical decision rule dedicated to this population. For instance, De Wit et al. derived the FALLS decision rule from a prospective multicentric study carried out in Canada and in the USA [22]. This rule has been recently prospectively validated [23]. This rule indicates that head CT scan may not be required if 1) patients did not hit their head when they fell, 2) no amnesia of the fall 3) no new abnormality on neurological examination, and 4) the Clinical Frailty Scale Score is < 5. Despite its low specificity (20.3%) the Falls Decision Rule might safely avoid head CT scans in 20% of patients. Interestingly, neither anti-platelets nor anticoagulants are mentioned in this rule.

The main strength of our study resides in its prospective nature, enabling an accurate description of clinical practices in usual care settings. However, in the absence of patients’ follow-up after 48 hours, the assessment of head CT scan may be questionable, as some patients may have had a head CT scan after the follow-up period. Nevertheless, this is limited by the fact that national guidelines recommend a CT scan prescription within 8 hours after the trauma [13]. Moreover, the prevalence of traumatic ICH may have been underestimated, since not all patients received a CT scan. Furthermore, the small sample sizes reported for certain regions (e.g., Bourgogne, Normandie) may limit the power of our statistical analyses.

## Conclusions

Head CT scan use in patient presenting to EDs with head injuries after ground-level falls is highly variable. High rate of head CT scan use is not correlated with high traumatic intracranial hemorrhage yield rate. The prescription of appropriate head CT scan is low in this population. These results support the use of a clinical decision rule dedicated to this population in order to standardize our practices.

## Supporting information

S1 Table Head CT scan use following Emergency Departments’ characteristics. (DOCX)

S2 Table Head CT scan use following patients’ characteristics. (DOCX)

S3 Table Predictive factors associated with head CT scan use at the Emergency Department in patients with ground-level fall-related minor head trauma presenting with Glasgow Coma scale score 15 neither focal neurologic sign nor anticoagulant using mixed logistic regression. (DOCX)

S4 Table Predictive factors associated with head CT scan use at the Emergency Department in patients with ground-level fall-related minor head trauma presenting with Glasgow Coma scale score 15 without focal neurologic sign using mixed logistic regression. (DOCX)

S5 Table Predictive factors associated with head CT scan use at the Emergency Department in patients with ground-level fall-related minor head trauma presenting with Glasgow Coma scale score 15 neither focal neurologic sign nor anticoagulant using mixed logistic regression. (DOCX)
