# Supplementary material for: Variability in head computed tomography use for minor head injury after ground-level falls in the emergency department: A subanalysis of EPI-TC study
Source: PLoS One. 2026 Jan 2;21(1):e0334541. doi: 10.1371/journal.pone.0334541 (PMC12758682; doi:10.1371/journal.pone.0334541)
Supplement: S1 Table — (DOCX) [file pone.0334541.s001.docx]

**Table S1. Head CT scan use following Emergency Departments’ characteristics**

|  | Overall | Head CT scan performed | No Head CT scan performed | p-value |
| --- | --- | --- | --- | --- |
|  | N = 631 | N = 409 | N = 222 |  |
| **Type of institution** |  |  |  | 0.67 |
| **University Hospital** | 345 | 221 (54) | 124 (55.9) |  |
| **No affiliated University Hospital** | 286 | 188 (46) | 98 (44.1) |  |
|  |  |  |  |  |
| **Emergency department admissions per day** | |  |  | 0.1 |
| **<100** | 142 | 101 (24.7) | 41 (18.4) |  |
| **100-200** | 343 | 211 (51.6) | 132 (59.5) |  |
| **>200** | 146 | 97 (23.7) | 49 (22.1) |  |
|  |  |  |  |  |
| **Onsite neurosurgery units** | 298 | 197 (48.2) | 101 (45.5) | 0.52 |
