# Supplementary material for: Variability in head computed tomography use for minor head injury after ground-level falls in the emergency department: A subanalysis of EPI-TC study
Source: PLoS One. 2026 Jan 2;21(1):e0334541. doi: 10.1371/journal.pone.0334541 (PMC12758682; doi:10.1371/journal.pone.0334541)
Supplement: S2 Table — (DOCX) [file pone.0334541.s002.docx]

**Table S2. Head CT scan use following patients’ characteristics**

|  | **Overall** | **Head CT scan performed** | **No Head CT scan performed** | **p-value** |
| --- | --- | --- | --- | --- |
|  | **N = 631** | **N = 409** | **N = 222** |  |
|  |  |  |  |  |
| Age, median (IQ1-IQ3) | 79 (63-88) | 67 (48-88) | 53 (28-75) | <0.001 |
| Sex, female | 383 (60.7) | 250 (61.1) | 133 (59.9) | 0.8 |
|  |  |  |  |  |
| **Place of residence** |  |  |  | 0.002 |
| Community-dwelling | 483 (76.6) | 299 (73.6) | 184 (83.6) |  |
| Nursing Homes | 137 (21.7) | 105 (25.9) | 32 (14.5) |  |
| Homelessness | 6 (1.0) | 2 (0.5) | 4 (1.8) |  |
|  |  |  |  |  |
| **Antiplatelets** | 154 (24.4) | 139 (34.7) | 15 (6.7) | <0.001 |
| **Anticoagulants** | 141(22.4) | 134 (33.5) | 7 (3.2) | <0.001 |
|  |  |  |  |  |
| **Post head trauma symptoms** | |  |  |  |
| Amnesia | 39 (6.2) | 32 (8.7) | 7 (3.3) | 0.01 |
| Loss of consciousness | 69 (10.9) | 56 (15.3) | 13 (6.2) | 0.001 |
| Confusion | 72 (11.4) | 61 (16.7) | 11 (5.2) | <0.001 |
| Headache | 93 (14.7) | 63 (17.2) | 30 (14.2) | 0.34 |
| Seizure | 3 (0.5) | 2 (0.5) | 1 (0.4) | 0.9 |
| Vomiting | 37 | 28 (7.7) | 9 (4.3) | 0.1 |
|  |  |  |  |  |
| **Clinical findings at the ED** |  |  |  |  |
| Coma Glasgow Scale Score |  |  |  | 0.1 |
| *15* | 587 (93.0) | 372 (90.1) | 215 (96.8) |  |
| *14* | 37 (5.9) | 30 (7.3) | 7 (3.1) |  |
| *13* | 7 (1.1) | 7 (1.7) | 0 |  |
| Visible head impact location |  |  |  | 0.15 |
| *None* | 155 (24.6) | 99 (24.6) | 56 (25.6) |  |
| *Facial* | 104 (16.5) | 66 (16.3) | 38 (17.4) |  |
| *Frontal* | 153 (24.3) | 94 (23.1) | 59 (26.9) |  |
| *Temporal-parietal-occipital* | 172 (27.3) | 113 (27.8) | 59 (26.9) |  |
| *Multiple* | 41 (6.5) | 34 (8.4) | 7 (3.2) |  |
| Focal neurological signs | 16 (2.5) | 15 (3.7) | 1 (0.5) | 0.02 |
| Pupillary abnormalities | 6 (1.0) | 5 (1.3) | 1 (0.5) | 0.35 |
| Basal skull fracture signs | 20 (3.2) | 17 (4.2) | 3 (1.4) | 0.06 |
|  |  |  |  |  |
| **Fall precipitating factor** |  |  |  | 0.003 |
| Environmental | 323 (51.2) | 183 (44.7) | 140 (63.1) |  |
| Syncope | 45 (7.1) | 39 (11.0) | 6 (2.7) |  |
| Faintness or vertigo | 81 (12.8) | 47 (11.5) | 34 (15.3) |  |
| Alcohol intoxication | 29 (4.6) | 19 (38.8) | 10 (4.5) |  |
| Others | 7 (1.1) | 3 (0.7) | 4 (1.8) |  |
