# Supplementary material for: Variability in head computed tomography use for minor head injury after ground-level falls in the emergency department: A subanalysis of EPI-TC study
Source: PLoS One. 2026 Jan 2;21(1):e0334541. doi: 10.1371/journal.pone.0334541 (PMC12758682; doi:10.1371/journal.pone.0334541)
Supplement: S3 Table — (DOCX) [file pone.0334541.s003.docx]

**Table S3. Predictive factors associated with head CT scan use at the Emergency Department in patients with ground-level fall-related minor head trauma presenting with Glasgow Coma scale score 15 neither focal neurologic sign nor anticoagulant using mixed logistic regression**

|  | Odds ratio | CI 95 % | p-value |
| --- | --- | --- | --- |
|  |  |  |  |
| Age, per year | 1.0 | 1.0-1.1 | 0.04 |
|  |  |  |  |
| Antiplatelets | 21.7 | 9.4 – 50.1 | <0.001 |
|  |  |  |  |
| Fall precipitating factor |  |  |  |
| Syncope | 7.9 | 2.2 – 27.5 | 0.001 |
| Faintness or vertigo | 1.0 | 0.5 - 2.1 | 0.91 |
| Alcohol intoxication | 7.2 | 1.7 – 29.8 | 0.006 |
| Others | 0.7 | 0.1 – 19.5 | 0.84 |
|  |  |  |  |
| Clinical findings at the ED |  |  |  |
| Amnesia | 5.5 | 1.5 - 19.7 | 0.008 |
| Loss of consciousness | 4.6 | 1.6 – 13.2 | 0.005 |
| Vomiting | 7.8 | 2.2 – 28.2 | 0.002 |
