# Supplementary material for: Variability in head computed tomography use for minor head injury after ground-level falls in the emergency department: A subanalysis of EPI-TC study
Source: PLoS One. 2026 Jan 2;21(1):e0334541. doi: 10.1371/journal.pone.0334541 (PMC12758682; doi:10.1371/journal.pone.0334541)
Supplement: S4 Table — (DOCX) [file pone.0334541.s004.docx]

**Table S4. Predictive factors associated with head CT scan use at the Emergency Department in patients with ground-level fall-related minor head trauma presenting with Glasgow Coma scale score 15 without focal neurologic sign using mixed logistic regression**

|  | Odds ratio | CI 95 % | p-value |
| --- | --- | --- | --- |
|  |  |  |  |
| Age, per year | 1.0 | 1.0-1.1 | 0.04 |
|  |  |  |  |
| Antiplatelets | 28.5 | 11.6 - 69.9 | <0.001 |
| Anticoagulants | 69.9 | 19.4 - 251 | <0.001 |
|  |  |  |  |
| Fall precipitating factor |  |  |  |
| Syncope | 8.8 | 2.4 – 31.9 | 0.001 |
| Faintness or vertigo | 1.0 | 0.4 - 2.2 | 0.98 |
| Alcohol intoxication | 6.2 | 1.4 – 27.0 | 0.01 |
| Others | 0.6 | 0.2 – 19.1 | 0.78 |
|  |  |  |  |
| Clinical findings at the ED |  |  |  |
| Amnesia | 3.9 | 1.2 - 13.2 | 0.03 |
| Loss of consciousness | 5.0 | 1.7 - 14.9 | 0.004 |
| Vomiting | 7.6 | 2.1 – 27.2 | 0.002 |
