# Supplementary material for: Variability in head computed tomography use for minor head injury after ground-level falls in the emergency department: A subanalysis of EPI-TC study
Source: PLoS One. 2026 Jan 2;21(1):e0334541. doi: 10.1371/journal.pone.0334541 (PMC12758682; doi:10.1371/journal.pone.0334541)
Supplement: Attachment — Submitted filename: Response to Reviewers.docx [file pone.0334541.s006.docx]

September 12, 2025

To Dr. Jan Chrusciel, Academic Editor,

Plos One

Ref: PONE-D-25-25181e

Title: Variation in the use of head CT scan in patients attending the emergency department after ground-level fall-related minor head injury

Dear Editor,

Thank you for oﬀering us the opportunity to revise our manuscript “Variation in the use of head CT scan in patients attending the emergency department after ground-level fall-related minor head injury” and resubmit it for publication in “Plos One”. We have revised the paper according to the Editorial and Reviewer’s comments. In this letter, we address the changes made point by point.

We would like to thank the Reviewers for his/her comments, which have given us the opportunity to further improve our manuscript. We trust that you will find our revisions acceptable and look forward to hearing from you.

Yours sincerely,

Xavier DUBUCS

**Editorial comments.**

Journal Requirements:

When submitting your revision, we need you to address these additional requirements.

1. Please ensure that your manuscript meets PLOS ONE's style requirements, including those for file naming. The PLOS ONE style templates can be found at

<https://journals.plos.org/plosone/s/file?id=wjVg/PLOSOne_formatting_sample_main_body.pdf> and [https://journals.plos.org/plosone/s/file?id=ba62/PLOSOne_formatting_sample_title_authors_affiliations.pdf](https://track.editorialmanager.com/CL0/https:%2F%2Fjournals.plos.org%2Fplosone%2Fs%2Ffile%3Fid=ba62%2FPLOSOne_formatting_sample_title_authors_affiliations.pdf/1/010f0198844311f2-a516677c-1893-4b0c-aed1-b9c1592a754d-000000/rJijfmEWkbWnEwNp-Iz_W2-aZIrR0Azo4KhCsowQBdo=223)

**Response:**

**We have made all necessary changes in accordance with these recommendations.**

2. In the ethics statement in the Methods, you have specified that verbal consent was obtained. Please provide additional details regarding how this consent was documented and witnessed, and state whether this was approved by the IRB.

**Response:**

**We have enhanced the clarity of the Ethics Statement section:**

**“According to French and European law on ethics, all included patients were informed that their anonymized data will be used for the study. The oral non-opposition of all patients was collected and a written information notice was given to each included patient explaining the objectives of the study and the legislation explaining this research. This non-opposition was documented in the patient's medical file. According to the French ethics and regulatory law (public health code) prospective studies based on the exploitation of usual care data don’t should be submit at an ethics committee (IRB) but they have to be declared or covered by reference methodology of the French National Commission for Informatics and Liberties (CNIL). Toulouse University Hospital signed a commitment of compliance to the reference methodology MR-004 of the French National Commission for Informatics and Liberties (CNIL). After evaluation and validation by the data protection officer and according to the General Data Protection Regulation (Regulation EU 2016/679 of the European Parliament and of the Council of 27 April 2016), this study completing all the criteria, it is register in the register of data study of the Toulouse University Hospital (number’s register: RnIPH 2022–78) and cover by the MR-004 (CNIL number: 2206723 v 0). This study was approved by Toulouse University Hospital and confirms that ethical requirements were totally respected in the above report.”**

3. Please include a complete copy of PLOS’ questionnaire on inclusivity in global research in your revised manuscript. Our policy for research in this area aims to improve transparency in the reporting of research performed outside of researchers’ own country or community. The policy applies to researchers who have travelled to a different country to conduct research, research with Indigenous populations or their lands, and research on cultural artefacts. The questionnaire can also be requested at the journal’s discretion for any other submissions, even if these conditions are not met. Please find more information on the policy and a link to download a blank copy of the questionnaire here: <https://journals.plos.org/plosone/s/best-practices-in-research-reporting>. Please upload a completed version of your questionnaire as Supporting Information when you resubmit your manuscript.

**Response:**

**This document has been completed.**

4. We note that the grant information you provided in the ‘Funding Information’ and ‘Financial Disclosure’ sections do not match.

When you resubmit, please ensure that you provide the correct grant numbers for the awards you received for your study in the ‘Funding Information’ section.

**Response:**

**This grant number has been added**

5. Thank you for stating in your Funding Statement:

[This study was supported by the French Emergency Medicine Research Network (Initiatives de Recherche aux Urgences [IRU group]) of the Société Française de Médecine d’Urgence (SFMU). The funder had no role in the design, data collection, data analysis, and reporting of this study.].

Please provide an amended statement that declares *all* the funding or sources of support (whether external or internal to your organization) received during this study, as detailed online in our guide for authors at <http://journals.plos.org/plosone/s/submit-now>.  Please also include the statement “There was no additional external funding received for this study.” in your updated Funding Statement.

Please include your amended Funding Statement within your cover letter. We will change the online submission form on your behalf.

**Response:**

**These sentences have been added.**

6. Thank you for stating the following in your manuscript:

[This study was supported by the French Emergency Medicine Research Network (Initiatives de Recherche aux Urgences [IRU group]) of the Société Française de Médecine d’Urgence (SFMU). The funder had no role in the design, data collection, data analysis, and reporting of this study.]

We note that you have provided funding information that is currently declared in your Funding Statement. However, funding information should not appear in the Acknowledgments section or other areas of your manuscript. We will only publish funding information present in the Funding Statement section of the online submission form.

Please remove any funding-related text from the manuscript and let us know how you would like to update your Funding Statement. Currently, your Funding Statement reads as follows:

[This study was supported by the French Emergency Medicine Research Network (Initiatives de Recherche aux Urgences [IRU group]) of the Société Française de Médecine d’Urgence (SFMU). The funder had no role in the design, data collection, data analysis, and reporting of this study.]

Please include your amended statements within your cover letter; we will change the online submission form on your behalf.

**Response:**

**This chapter has been removed from the manuscript and the cover letter has been modified to reflect the previous suggestions.**

7. In the online submission form, you indicated that [The data on which the results presented in the study are based are available on request from Xavier Dubucs.].

All PLOS journals now require all data underlying the findings described in their manuscript to be freely available to other researchers, either 1. In a public repository, 2. Within the manuscript itself, or 3. Uploaded as supplementary information.

This policy applies to all data except where public deposition would breach compliance with the protocol approved by your research ethics board. If your data cannot be made publicly available for ethical or legal reasons (e.g., public availability would compromise patient privacy), please explain your reasons on resubmission and your exemption request will be escalated for approval.

**Response:**

**We have changed our answers to these questions.**

8. When completing the data availability statement of the submission form, you indicated that you will make your data available on acceptance. We strongly recommend all authors decide on a data sharing plan before acceptance, as the process can be lengthy and hold up publication timelines. Please note that, though access restrictions are acceptable now, your entire data will need to be made freely accessible if your manuscript is accepted for publication. This policy applies to all data except where public deposition would breach compliance with the protocol approved by your research ethics board. If you are unable to adhere to our open data policy, please kindly revise your statement to explain your reasoning and we will seek the editor's input on an exemption. Please be assured that, once you have provided your new statement, the assessment of your exemption will not hold up the peer review process.

**Response:**

**I agree, if my article is accepted, to make my data freely available.**

9. One of the noted authors is a group or consortium [IRU EPI-TC group]. In addition to naming the author group, please list the individual authors and affiliations within this group in the acknowledgments section of your manuscript. Please also indicate clearly a lead author for this group along with a contact email address.

**Response:**

**Done**

10. We note that Figures 2 and S1 in your submission contain map images which may be copyrighted. All PLOS content is published under the Creative Commons Attribution License (CC BY 4.0), which means that the manuscript, images, and Supporting Information files will be freely available online, and any third party is permitted to access, download, copy, distribute, and use these materials in any way, even commercially, with proper attribution. For these reasons, we cannot publish previously copyrighted maps or satellite images created using proprietary data, such as Google software (Google Maps, Street View, and Earth). For more information, see our copyright guidelines: <http://journals.plos.org/plosone/s/licenses-and-copyright>.

**Response:**

**We have decided to remove these two figures because we are unable to provide copyright information.**

We require you to either (1) present written permission from the copyright holder to publish these figures specifically under the CC BY 4.0 license, or (2) remove the figures from your submission:

1. You may seek permission from the original copyright holder of Figures 2 and S1 to publish the content specifically under the CC BY 4.0 license.

We recommend that you contact the original copyright holder with the Content Permission Form (<http://journals.plos.org/plosone/s/file?id=7c09/content-permission-form.pdf>) and the following text:

“I request permission for the open-access journal PLOS ONE to publish XXX under the Creative Commons Attribution License (CCAL) CC BY 4.0 (<http://creativecommons.org/licenses/by/4.0/>). Please be aware that this license allows unrestricted use and distribution, even commercially, by third parties. Please reply and provide explicit written permission to publish XXX under a CC BY license and complete the attached form.”

Please upload the completed Content Permission Form or other proof of granted permissions as an "Other" file with your submission

In the figure caption of the copyrighted figure, please include the following text: “Reprinted from [ref] under a CC BY license, with permission from [name of publisher], original copyright [original copyright year].”

2. If you are unable to obtain permission from the original copyright holder to publish these figures under the CC BY 4.0 license or if the copyright holder’s requirements are incompatible with the CC BY 4.0 license, please either i) remove the figure or ii) supply a replacement figure that complies with the CC BY 4.0 license. Please check copyright information on all replacement figures and update the figure caption with source information. If applicable, please specify in the figure caption text when a figure is similar but not identical to the original image and is therefore for illustrative purposes only.

The following resources for replacing copyrighted map figures may be helpful:

USGS National Map Viewer (public domain): <http://viewer.nationalmap.gov/viewer/>
The Gateway to Astronaut Photography of Earth (public domain): <http://eol.jsc.nasa.gov/sseop/clickmap/>
Maps at the CIA (public domain): <https://www.cia.gov/library/publications/the-world-factbook/index.html> and [https://www.cia.gov/library/publications/cia-maps-publications/index.html](https://track.editorialmanager.com/CL0/https:%2F%2Fwww.cia.gov%2Flibrary%2Fpublications%2Fcia-maps-publications%2Findex.html/1/010f0198844311f2-a516677c-1893-4b0c-aed1-b9c1592a754d-000000/VMIhOaGF5rMpz4iWVtj_kVVO8OR6dj9jT7uO6Q35RNo=223)
NASA Earth Observatory (public domain): <http://earthobservatory.nasa.gov/>
Landsat: <http://landsat.visibleearth.nasa.gov/>
USGS EROS (Earth Resources Observatory and Science (EROS) Center) (public domain): [http://eros.usgs.gov/#](http://eros.usgs.gov/)
Natural Earth (public domain): <http://www.naturalearthdata.com/>

11. We notice that your supplementary figure is uploaded with the file type 'Figure'. Please amend the file type to 'Supporting Information'. Please ensure that each Supporting Information file has a legend listed in the manuscript after the references list.

**Response:**

**These changes have been made.**

If the reviewer comments include a recommendation to cite specific previously published works, please review and evaluate these publications to determine whether they are relevant and should be cited. There is no requirement to cite these works unless the editor has indicated otherwise. 

**Reviewers' comments:**

Reviewer's Responses to Questions

**Comments to the Author**

1. Is the manuscript technically sound, and do the data support the conclusions?

The manuscript must describe a technically sound piece of scientific research with data that supports the conclusions. Experiments must have been conducted rigorously, with appropriate controls, replication, and sample sizes. The conclusions must be drawn appropriately based on the data presented.

Reviewer #1: Partly

Reviewer #2: Partly

2. Has the statistical analysis been performed appropriately and rigorously?

Reviewer #1: I Don't Know

Reviewer #2: Yes

3. Have the authors made all data underlying the findings in their manuscript fully available?

The [PLOS Data policy](http://track.editorialmanager.com/CL0/http:%2F%2Fwww.plosone.org%2Fstatic%2Fpolicies.action%23sharing/1/010f0198844311f2-a516677c-1893-4b0c-aed1-b9c1592a754d-000000/VyiZaqP5ax4PZpcPNwynKI7CQE7ezCm_YR_ouc5oT3c=223) requires authors to make all data underlying the findings described in their manuscript fully available without restriction, with rare exception (please refer to the Data Availability Statement in the manuscript PDF file). The data should be provided as part of the manuscript or its supporting information, or deposited to a public repository. For example, in addition to summary statistics, the data points behind means, medians and variance measures should be available. If there are restrictions on publicly sharing data—e.g. participant privacy or use of data from a third party—those must be specified.

Reviewer #1: Yes

Reviewer #2: Yes

4. Is the manuscript presented in an intelligible fashion and written in standard English?

PLOS ONE does not copyedit accepted manuscripts, so the language in submitted articles must be clear, correct, and unambiguous. Any typographical or grammatical errors should be corrected at revision, so please note any specific errors here.

Reviewer #1: Yes

Reviewer #2: No

5. Review Comments to the Author

**Reviewer #1:**

Thank you for the opportunity to review this manuscript. The proposed topic highlights several important issues concerning a subject with significant consequences for patients. I hope these comments will help the authors.

Title:
The title does not specify that this is a secondary analysis of an already published article.

**Response:**

**Please find below a new proposal for the title:**

**Variability in head computed tomography use for minor head injury after ground-level falls in the emergency department: a subanalysis of EPI-TC study**

Abstract:
L 136: The described outcomes does not correspond to the study's outcomes.

**Response:**

**Indeed, there was some inaccuracy, we have harmonized this in the abstract, the introduction, and the Methods section.**

**« The aim of this study was to assess the variation in the use of head CT scan in patients attending EDs with ground-level fall-related minor head injury. Secondary objectives were: i) to measure the prescription rate of appropriated head CT scan, ii) to identify patients’ and EDs characteristics associated with head CT scan prescription iii) to explore potential correlation between head CT scan use and traumatic intracranial hemorrhage (ICH) yield rate in this population. »**

Introduction:
L 156: Reference 1 could be supplemented with a more recent article by Andrew I R Maas et al, "Traumatic brain injury: progress and challenges in prevention, clinical care, and research," The Lancet Neurology, 2022.

**Response:**

**We modified this reference.**

L 161: References 3 and 4 should be positioned in the previous sentence (L159).

**Response:**

**Done.**

L 169: Reference should be supplemented by Majdan et al, "Epidemiology of traumatic brain injuries in Europe: a cross-sectional analysis," The Lancet Public Health, 2016.

**Response:**

**Done.**

L 170: The publication by Mori et al, "Indications for Computed Tomography in Older Adult Patients With Minor Head Injury in the Emergency Department," Academic Emergency Medicine, 2020, is a more recent reference.

**Response:**

**We had this reference.**

Materials and Methods:
L 182: 71 EDs participated in the original study ("Head injuries in prehospital and Emergency Department settings: a prospective multicentre cross sectional study in France" by Dubucs et al., BMC Emergency Medicine, 2024), but only 63 are included in this study. What explains the difference? Did some centers not perform CT scans?

**Response:**

**As mentioned in the methods section, “in this sub-analysis, only patients sustaining a ground-level fall-related with minor head injury (defined as Glasgow Coma Scale (GCS) score ≥13 upon arrival) were included”. Thus, for eight centers, head injuries resulted from a mechanism other than ground-level falls. This inclusion criterion explains the difference in the number of centers between the main analysis and this subanalysis analysis.**

L 187 to 191: It would be preferable to cite those that are missing. It would be interesting to have a table by ED “Type of institution” meaning a mix of tables S1 and S2.

**Response**

**We are unable to cite the centers that were unable to participate, as this list is not official. It is estimated that approximately 100 centers are affiliated with this research network, although this number changes annually.**

**In order to better clarify the distribution of “type of institution by region,” and in accordance with your proposal, we have added a column to Table S1 specifying this information. Following the editor's comments, we have incorporated Table S1 into the manuscript: Table 2.**

L 211: What data were collected regarding the patient's hospital stay?

**Response:**

**As mentioned in the Data collection and study variables section, “… sociodemographic information, pre-injury use of antiplatelet and anticoagulant medications, fall mechanisms, symptoms experienced after head injury, and other relevant clinical data related to the patient’s stay at the ED and hospital.”.**

**Regarding the patients' hospital stay, only the performance of a head CT scan (primary outcome), as well as post-emergency department outcomes (discharge, hospital admission, or in-hospital death) were reported. We added this information in the manuscript.**

**“This included sociodemographic information, pre-injury use of antiplatelet and anticoagulant medications, fall precipitating factor, symptoms experienced after head injury, clinical examination at the ED and post-emergency outcome (discharge, hospital admission and in-hospital death). Urgent neurosurgical interventions occurring within 48 hours after admission were also reported.”**

Results:
L 251: In the original study, there were 636 patients who had a CT scan, but in this article, there are 631 patients included.

**Response:**

**This is because the inclusion criterion for this ancillary study is a ground-level fall associated with a minor head injury (defined as a Glasgow Coma Scale (GCS) score ≥13 upon arrival). The performance of a head CT scan was not the inclusion criterion.**

L 253: According to Table 1, the number of patients without post-TBI symptoms does not correspond.

**Response:**

**We added a new row to this table displaying the number of patients without any post-head injury symptoms. Since some patients may have multiple symptoms, this explains why the total number of symptoms exceeds the number of patients.**

L 256: The results of the study's second objective are not sufficiently highlighted. They only appear in Figure 3. The proportions of patients who received appropriate CT scan prescriptions according to traumatic ICH risk (high and intermediate) are missing, which is the secondary objective.

**Response:**

**This proportion is reported at the end of the first paragraph of the Results section. To improve clarity, we moved this information immediately after the data on head CT scan prescriptions:**

**“A head CT scan was performed in 409 patients (64.8%, CI95% 61.0-68.5) and a traumatic ICH was reported in 29 (7.1%, CI95% 4.8-10.0) of them. Among head CT scan prescriptions, 80 of them (19.6%, (CI95% 15.8-23.7) were classified as appropriated. Two patients required urgent neurosurgery. »**

L 259: What is the number of patients with unknown mechanism? This does not appear in the flow chart.

**Response:**

**In Table 1, we revised the term 'fall mechanism' to 'fall precipitating factor' to improve clarity. As shown in Table 1, the fall precipitating factors were unknown in 146 patients.**

L 261: Table 1: What do the missing values for focal neurological signs, pupillary abnormalities, and basal skull fracture signs represent? Table S3 provides the same information as Table 1 but presented more relevantly; it would be preferable to exchange them.

**Response:**

**As specified in the Statistical Analysis section, “missing data rates were reported for each variable, and no data imputation was performed”. The table presents the baseline characteristics of the patients in our cohort. Therefore, the number of missing data is indicated for each variable, where applicable. To maintain clarity, this information is not repeated in subsequent tables (e.g., Table S3). According to the editor's opinion, we can remove this information from Table 1, but from our point of view, this information is important to keep.**

L 264: Table S1: What are the arguments for stating that at the regional level, patient characteristics are similar? In this same table, would it be possible to add a column with the "Type of institution" variable? Furthermore, it is difficult to establish a link between heterogeneity in CT scan prescriptions at the regional level and heterogeneity in the practices of clinicians working in emergency departments

**Reponse:**

**Thank you for your question. Indeed, in this first version, we did not sufficiently explain why a region-wide analysis was carried out. In France, there are regional colleges of emergency physicians that offer annual training conferences and continuing education courses. These courses are not standardized at the national level. National guidelines must be followed by all emergency physicians, but there may be some variability in practices related to the regional training courses offered. In addition, the regional analysis allows us to obtain a sufficient number of patients to better study this variability in prescribing. We have added this clarification to the article.**

**“In France, there are regional colleges of emergency physicians that offer annual training conferences and continuing education courses. These courses are not standardized at the national level. National guidelines must be followed by all emergency physicians, but there may be some variability in practices related to the regional training courses offered.”**

**In lines with your previous comment, we added a column with “Type of Institution” in the table S1. Following the editor's comments, we have incorporated Table S1 into the manuscript: Table 2.**

L 266: Figure 2 - the p-value does not appear on the map. The regional level lacks granularity. The type of institution by region should be displayed.

**Response:**

**Unfortunately, following review by the editors, due to copyright issues, we have removed this figure. All of this information, including the “type of institution,” is presented in Table S1. We therefore propose to incorporate Table S1 into the manuscript: Table 2.**

L 273: Table 2 – legend is missing

**Response:**

**We added this information.**

L 278: How is this correlation determined?

**Thank you for your comment. The estimation of this correlation was indeed questionable. In this first version, we deduced the absence of correlation based on the visual appearance of the figure. To be more precise in our analysis, we have added an evaluation of this correlation in this version by calculating Spearman's correlation coefficient (in order to comply with the conditions of use). To improve the clarity of the article, we have added this analysis to the objectives, method, and results.**

**In the introduction:**

**“Secondary objectives were: i) to measure the prescription rate of appropriated head CT scan, ii) to identify patients’ and EDs characteristics associated with head CT scan prescription iii) to explore potential correlation between head CT scan use and traumatic ICH yield rate in this population.”**

**In the methods:**

**“The proportion of appropriate use of head CT scan was calculated based on the number of head CT scan prescriptions. The correlation between the prevalence of traumatic ICH yield and head CT scan prescription rates -both overall and appropriate- was assessed using Spearman’s rank correlation coefficient.”**

**In the results:**

**“Among head CT scan prescriptions, 80 of them (19.6%, (CI95% 15.8-23.7) were classified as appropriated. No significant correlation was found between overall head CT scan use (Spearman’s correlation coefficient: 0.22, p=0.46) or appropriate head CT scan use (Spearman’s correlation coefficient: 0.06, p=0.86) and traumatic ICH rate.”**

L 281: No p-value on the graph in Figure 3. The regional level does not facilitate reading of results because some regions included few patients (Bourgogne FC, Normandie) and strongly impact the results shown in Figure 3. Furthermore, is the proportion of appropriate head CT scan prescriptions based on the total patients admitted to the ED after ground-level fall-related minor head injury or on the proportion of head CT scan use?

**Response:**

**Indeed, the small sample sizes in certain regions represent a limitation of our analysis. This limitation has been addressed in the discussion section. Furthermore, in response to your previous comment, we refined our correlation calculations by using Spearman’s rank correlation coefficient, which is suited for the analysis of small and potentially non-normally distributed samples.**

**“Furthermore, the small sample sizes reported for certain regions (e.g., Bourgogne, Normandie) may limit the power of our statistical analyses.”**

**We did not include the p-value in the figure so as not to overload the presentation of the results. We specified this p-value in the sentence introducing the figure.**

**“No statistically significant association was observed between the prevalence of traumatic ICH and regional distribution (p = 0.29) (Fig. 2)”**

**The proportion of appropriate head CT scan was based on head CT scan use. We added this precision in the statistical section:**

**“The proportion of appropriate use of head CT scan was calculated based on the number of head CT scan prescriptions.”**

Discussion:
L 296: Does the heterogeneity concern CT use among patients admitted to the ED after ground-level fall-related minor head injury? It appears that even for appropriate prescriptions, there is no correlation with ICH incidence.
L 298: "Our study also suggests that this variation may be explained by patients' characteristics." I do not understand this suggestion - CT scan prescription recommendations are based on patient characteristics.

**Response:**

**In this new version, we have improved the clarity of the beginning of this discussion and corrected certain misinterpretations. There was an error at the beginning of the chapter explaining the misunderstanding: « Our study suggests that this variation is not explained by patients’ characteristics ». In addition, the term “heterogeneity” was confusing, so we replaced it with the correct term “correlation.” We then reorganized the entire discussion to better align with our objectives.**

L 301: I completely agree with you that the main problem concerns implementation science, as explained by Cabana MD et al. "Why don't physicians follow clinical practice guidelines? A framework for improvement." JAMA. 1999, or Tucker et al. "Implementation Science: Application of Evidence-Based Practice Models to Improve Healthcare Quality." Worldviews Evid Based Nurs. 2021. The regional level does not seem relevant to explain heterogeneity. It is probably more at the individual level and how each clinician balances their clinical experience with recommendations issued by scientific societies, which are two of the pillars of EBM.

**Response:**

**We have added these references to our discussion. Indeed, the interpretation of our analyses at the regional level has limitations. In light of your previous comments, the arguments and limitations of this analysis have been incorporated into the manuscript.**

L 315: A high rate of CT prescription is not associated with ICH prevalence, but is a high rate of appropriate prescriptions associated with a high rate of ICH?

**Response:**

**In line with the response to your previous comment:**

**“Among head CT scan prescriptions, 80 of them (19.6%, (CI95% 15.8-23.7) were classified as appropriated. No significant correlation was found between overall head CT scan use (Spearman’s correlation coefficient: 0.22, p=0.46) or appropriate head CT scan use (Spearman’s correlation coefficient: 0.06, p=0.86) and traumatic ICH yield rate.”**

Response:
L 325: Where does this appear in your results?

**Response:**

**Following the reformatting of the discussion, we have also removed this section, which is presented in the results.**

L 349: This statement is particularly true for elderly patients who are not prioritized for examinations like CT scans and who may have them at a distance from the trauma. This data is particularly important to highlight given the mean age of the population in this study.

**Response:**

**Yes, indeed, that is why we considered it important to mention it in the limits of this study.**

**Reviewer #2:**

This is a multicenter cross-sectional study done in France to evaluate the variations of CTH use in patients who sustained a GLF-related head injury. The secondary objective was to measure the rate of appropriate CTH orders and to identify characteristics associated with CTH orders

Major comments
1. Need clarification on this sentence in the beginning of results: "A head CT scan was performed in 409 patients (64.8%, CI95% 61.0-68.5) and a traumatic ICH was reported in 29 (4.6%, CI95% 3.1-6.5) of them." is 4.6% out of 631 patients in the study or out of 409 patients who received CT? I am reading it as ICH yield on all CTs ordered so it should be 7.1% (29/409). In addition, Table 2 is reporting ICH prevalence out of the total of 631 patients (cohort) as 7.1%, which is confusing here. Need to reconcile those percentages

**Response:**

**Thank you for your comment. The presentation of our results lacked clarity. We have now reported the prevalence of intracranial hemorrhage (ICH) among patients who underwent scanning. As you pointed out, this approach is indeed more relevant, given that the presence of ICH cannot be formally excluded in patients who did not receive a scan. We have revised the relevant sections and included this clarification in the Statistical Analysis** **section.**

**“The prevalence of traumatic ICH was calculated among patients who underwent a head CT scan.”**

2. In discussion, author concluded that : "This heterogeneity was not associated with significant difference of traumatic ICH prevalence." This is a bit confusing. traumatic ICH is there whether a CTH is ordered or not. In other words, for patients in the cohort who did not received a CT, they might have a traumatic ICH even if they didn't get the CTH, and would still contribute to the prevalence technically but it's just not known. Should consider changing that sentence to "traumatic ICH yield." Same apply to Conclusion and implications and the rest of the paper.

**Response:**

**Indeed, following the previous reviewer’s comments, we removed this sentence, as the term "heterogeneity" was not appropriate. Regarding the prevalence of traumatic ICH, in response to your previous comment, we have clarified how it was calculated. We also propose to address this point in the limitations section of our study**

**“Moreover, the prevalence of traumatic ICH may have been underestimated, since not all patients received a CT scan”**

**This nuance regarding "traumatic ICH yield" has been addressed in the Discussion section to improve clarity, as well as in the "Conclusion and Implications".**

**«Conclusions**

**Head CT scan use in patient presenting to EDs with head injuries after ground-level falls is highly variable. High rate of head CT scan use is not correlated with high traumatic intracranial hemorrhage yield rate. The prescription of appropriate head CT scan is low in this population. These results support the use of a clinical decision rule dedicated to this population in order to standardize our practices.”**

3. Authors use "appropriate CTH orders" numerously throughout the paper. It is assumed that it's any CTH ordered that concordant with National Guidelines (Study design and Settings). It might be good to define what is considered appropriate or inappropriate for this study since it's not explicitly stated.

**Response:**

**We added a clarification in the Outcome Measures section regarding the definition of the term "appropriate."**

**“Outcome measures**

**The main outcome was non-contrast head CT scan performed during ED stay. The secondary outcome was appropriated head CT scan defined by the French National Guidelines published in 2022. Hence, head CT scan prescription was classified as appropriate if patients undergoing head CT scan showed intermediate or high risk of traumatic ICH (12). Head CT scan prescription in mild TBI patients across French EDs is determined by the National Guidelines published in 2012 and updated in 2022 (12, 13). These guidelines recommend a head CT scan for mild TBI patients at intermediate risk (patients aged ≥65 years old with antiplatelet therapy, Glasgow Coma Scale (GCS) score <15 two hours after the trauma with a suspected intoxication, that sustained high-energy trauma, or with amnesia ≥30 minutes after the trauma) within 8 hours after being admitted to the ED. In patients presenting a high risk of traumatic ICH (hemostasis disorders, suspected basilar or cranial skull fracture, GCS <15 two hours after the trauma without intoxication, >1 vomiting episode, post-traumatic seizures, focal neurological deficit) a head CT scan is required within the first hour. “**

Minor comments
1. Recommend going back to review spelling and grammar for the whole paper: Line 175 "The secondary objective was to measure the
prescription rate of appropriated head CT scan and to identify patients’ and EDs characteristic"

**Response:**

**Indeed, there were spelling and grammar errors. We had the article proofread by a native English speaker. All corrections are highlighted in yellow in the article.**

2. Coma Glasgow Scale Score in Table 1 should say Glasgow Coma Scale Score

**Response:**

**Done. This change has been made in the other tables.**

6. PLOS authors have the option to publish the peer review history of their article ([what does this mean?](https://track.editorialmanager.com/CL0/https:%2F%2Fjournals.plos.org%2Fplosone%2Fs%2Feditorial-and-peer-review-process%23loc-peer-review-history/1/010f0198844311f2-a516677c-1893-4b0c-aed1-b9c1592a754d-000000/OjSBWFPBZRFyRqF1_2-r1-knf4bbHZozGjztu7gHR38=223)). If published, this will include your full peer review and any attached files.

If you choose “no”, your identity will remain anonymous but your review may still be made public.

**Do you want your identity to be public for this peer review?** For information about this choice, including consent withdrawal, please see our [Privacy Policy](https://track.editorialmanager.com/CL0/https:%2F%2Fwww.plos.org%2Fprivacy-policy/1/010f0198844311f2-a516677c-1893-4b0c-aed1-b9c1592a754d-000000/G-GwOwexxcpqyInRjY2wcqGcsp4953pJ9wUP6qQgv2c=223).

Reviewer #1: No

Reviewer #2: No
